# Pretransplant Serum Uromodulin and Its Association with Delayed Graft Function Following Kidney Transplantation—A Prospective Cohort Study

**DOI:** 10.3390/jcm10122586

**Published:** 2021-06-11

**Authors:** Stephan Kemmner, Christopher Holzmann-Littig, Helene Sandberger, Quirin Bachmann, Flora Haberfellner, Carlos Torrez, Christoph Schmaderer, Uwe Heemann, Lutz Renders, Volker Assfalg, Tarek M. El-Achkar, Pranav S. Garimella, Jürgen Scherberich, Dominik Steubl

**Affiliations:** 1 Department of Nephrology, Klinikum rechts der Isar, Technical University of Munich, 81675 Munich, Germany; christopher.holzmann-littig2@mri.tum.de (C.H.-L.); h.sandberger@gmx.net (H.S.); quirin.bachmann@tum.de (Q.B.); flora.haberfellner@tum.de (F.H.); carlos.at@live.de (C.T.); Christoph.Schmaderer@mri.tum.de (C.S.); Uwe.Heemann@mri.tum.de (U.H.); lutz.renders@tum.de (L.R.); dominik.steubl@gmx.de (D.S.); 2 Transplant Center, University Hospital Munich, Ludwig-Maximilians-University (LMU), 81377 Munich, Germany; 3 Department of Surgery, Klinikum rechts der Isar, Technical University of Munich, 81675 Munich, Germany; Volker.assfalg@tum.de; 4 Department of Medicine, Division of Nephrology, Indiana University School of Medicine, Indianapolis, IN 46202-5188, USA; telachka@iu.edu; 5 Division of Nephrology-Hypertension, University of California San Diego, San Diego, CA 92093-9111, USA; pgarimella@health.ucsd.edu; 6 Department of Nephrology and Clinical Immunology, Klinikum München-Harlaching, Teaching Hospital of the Ludwig-Maximilians-University, 81545 Munich, Germany; j.scherberich@web.de

**Keywords:** uromodulin, Tamm-Horsfall-protein, kidney transplantation, delayed graft function, ischemia-reperfusion injury

## Abstract

Delayed graft function (DGF) following kidney transplantation is associated with increased risk of graft failure, but biomarkers to predict DGF are scarce. We evaluated serum uromodulin (sUMOD), a potential marker for tubular integrity with immunomodulatory capacities, in kidney transplant recipients and its association with DGF. We included 239 kidney transplant recipients and measured sUMOD pretransplant and on postoperative Day 1 (POD1) as independent variables. The primary outcome was DGF, defined as need for dialysis within one week after transplantation. In total, 64 patients (27%) experienced DGF. In multivariable logistic regression analysis adjusting for recipient, donor and transplant associated risk factors each 10 ng/mL higher pretransplant sUMOD was associated with 47% lower odds for DGF (odds ratio (OR) 0.53, 95% confidence interval (95%-CI) 0.30–0.82). When categorizing pretransplant sUMOD into quartiles, the quartile with the lowest values had 4.4-fold higher odds for DGF compared to the highest quartile (OR 4.41, 95%-CI 1.54–13.93). Adding pretransplant sUMOD to a model containing established risk factors for DGF in multivariable receiver-operating-characteristics (ROC) curve analysis, the area-under-the-curve improved from 0.786 [95%-CI 0.723–0.848] to 0.813 [95%-CI 0.755–0.871, *p* = 0.05]. SUMOD on POD1 was not associated with DGF. In conclusion, higher pretransplant sUMOD was independently associated with lower odds for DGF, potentially serving as a non-invasive marker to stratify patients according to their risk for developing DGF early in the setting of kidney transplantation.

## 1. Introduction

Delayed graft function (DGF), commonly defined as need for dialysis within the first week after kidney transplantation, affects around 25–50% of patients, and is associated with a higher risk for acute rejection episodes and reduced long-term graft survival [1,2,3,4]. DGF presents histologically mainly as severe ischemia-reperfusion injury (IRI) with inflammatory tubular damage [5]. IRI triggers a long-term inflammation leading to interstitial fibrosis and tubular atrophy and reduces overall graft survival [6,7,8]. Therefore, understanding and potentially targeting the pathophysiology of IRI might improve long-term kidney graft survival [9]. However, measures to ameliorate IRI and markers predicting DGF before transplantation are scarce and still have limited diagnostic value [10].

Uromodulin (also known as Tamm-Horsfall protein), is a kidney derived glycoprotein with a molecular weight of around 100 kDa, exclusively expressed by epithelial cells of the thick ascending limb of the loop of Henle and the distal tubule [11,12,13]. The molecule is secreted both into the urine as well as the interstitium and circulation [14,15,16]. Thereby, interstitial uromodulin largely corresponds to serum concentrations in different forms of kidney disease [16]. Uromodulin is encoded by the UMOD gene, and mice lacking the UMOD gene showed more inflammation and tubular injury compared to wild type following renal IRI. In addition, they also demonstrate a greater necrotic and inflammatory phenotype of cell death rather than apoptotic, suggesting that interstitial uromodulin may have immunomodulating and anti-inflammatory capacities [17,18,19]. Uromodulin deficiency is also associated with delayed and in part incomplete kidney recovery following renal IRI in mice [14]. These data suggest that higher serum uromodulin (sUMOD) in the acute phase of kidney transplantation may be protective against IRI. Furthermore, higher sUMOD post-transplant is associated with lower risk for long-term kidney transplant failure [20,21]. However, the role of sUMOD in the early setting of transplantation and IRI remains to be investigated.

Here, we propose that recipient’s sUMOD plays an important role in the recovery from IRI after kidney transplantation, and thus sUMOD might be of predictive value for the incidence of DGF after kidney transplantation. In this study we evaluated recipient’s sUMOD pretransplant and on postoperative Day 1 (POD1) as a marker for prediction/early detection of DGF in kidney transplant recipients.

## 2. Materials and Methods

### 2.1. Study Participants and Study Design

In this single-center prospective observational cohort study, we recruited 239 patients undergoing kidney or combined kidney-pancreas transplantation following deceased or living donation in our tertiary care hospital. All patients who were able to provide informed consent were included in the study. Local institutional review boards of the Technical University of Munich, Germany approved the study methods. The study adheres to the declaration of Helsinki and the declaration of Istanbul.

### 2.2. Exposure

Serum samples for measuring sUMOD in the recipients were obtained 24 h prior to kidney transplantation in living organ donations and up to 5 h pretransplant in deceased donations, again on the first postoperative day (POD1) and subsequent time points later. Since all patients were hospitalized during the sample collection and no patient withdrew from the study, no patients were lost to follow-up for the primary endpoint (see below).

The samples were stored at −80 °C until they were thawed. sUMOD analyses were performed in singlicate using a commercial enzyme-linked immunosorbent assay (ELISA, Euroimmun, Medizinische Labordiagnostika AG, Lübeck, Germany) based on the manufacturer’s instructions. This assay is based on a colorimetric sandwich immunoassay using a polyclonal antibody against human uromodulin as the capture antibody and a biotinylated polyclonal antibody against human uromodulin as the detection antibody. Quality characteristics of the ELISA are as follows: intra-assay coefficient of variation 1.8–3.2%, inter-assay coefficient of variation 6.6–7.8%, mean linearity recovery 97%, and lower limit of detection 2.0 ng/mL.

### 2.3. Outcomes

The primary outcome was DGF, defined as the need for more than one dialysis within one week after transplantation as has been defined in prior clinical studies [22,23]. For example, one dialysis due to potassium lowering was not considered as DGF. Notably, in our tertiary center we avoid pretransplant dialysis to reduce cold ischemia time whenever reasonable, which leads to postoperative dialysis for hyperkalemia in some cases.

### 2.4. Statistical Analysis

We describe the population using mean (± standard deviation) for continuous variables and number with percentages for binary and categorical variables.

Multivariable logistic regression models were used to evaluate the association of sUMOD pretransplant and on POD1 as independent variables and DGF as the dependent variable. We applied a series of nested models: (i) unadjusted; (ii) adjusted for age, body-mass index (BMI) and dialysis vintage; (iii) Model 1 plus serum creatinine on POD1 (“Model 2”; we adjusted serum creatinine on POD1 as it appears to be an important variable for the decision to apply kidney replacement therapy postoperatively); (iv) Model 2 plus cold ischemia time (CIT), living vs. deceased donor transplantation, and expanded criteria donors (ECD) vs. standard criteria donors (“Model 3”). ECD are donors that are either older than 60 years, or 50–59 years old and meet at least two of the following criteria: cerebrovascular death, history of hypertension, and/or last serum creatinine greater than 1.5 mg/dL [24]. Due to the number of endpoints, we limited the analysis to these co-variables. Of note, we use the ECD classification for the adjustment because it covers donor age, donor serum creatinine and the donor cardiovascular cause of death. All variables were selected based on their clinical relevance for the outcomes of interest and are known risk factors for the primary endpoint DGF [2,5,25]. We performed multivariable receiver-operating-characteristic (ROC) curve analyses to evaluate the diagnostic value of preoperative sUMOD in addition to established risk factors (recipient age and BMI, dialysis vintage, CIT, deceased vs. living donation, ECD, “Model A”) for the prediction of DGF (“Model B”). All analyses were conducted using R, version 3.5.1 (R Core Team (2018), Vienna, Austria).

## 3. Results

### 3.1. Population Characteristics

The mean age of the cohort was 51 ± 14 years, 31.4% were female, 90 (37.7%) received an organ from a living donor, 79 (33.1%) had cardiovascular disease at baseline. Mean serum creatinine concentration was 6.0 ± 2.3 mg/dL on POD1. Demographics of the entire cohort and stratified by preoperative sUMOD quartiles are shown in Table 1.

A total of 64 (26.8%) renal allograft recipients experienced DGF. Patients who experienced DGF were older, more often male, had a higher BMI and a greater prevalence of cardiovascular disease (Table 2). The time on dialysis before transplantation (dialysis vintage) was significantly longer in recipients who developed DGF (2208 ± 1456 days vs. 1321 ± 1331, *p* < 0.001). The mean serum creatinine on POD1 was significantly higher in patients with DGF (7.1 ± 2.4 mg/dL vs. 5.6 ± 2.1 mg/dL, *p* < 0.001). Referring to donor characteristics, kidney transplants with subsequent DGF were derived from donors who were more often male, had a higher BMI, a higher prevalence of diabetes and had a significantly higher serum creatinine before donation. Furthermore, cold and warm ischemia time were significantly longer for donor kidneys who developed DGF. Further information on DGF vs. non-DGF patients can be found in Table 2.

### 3.2. Course of sUMOD during the Transplant Process and Short Term Follow Up

**Figure 1:**
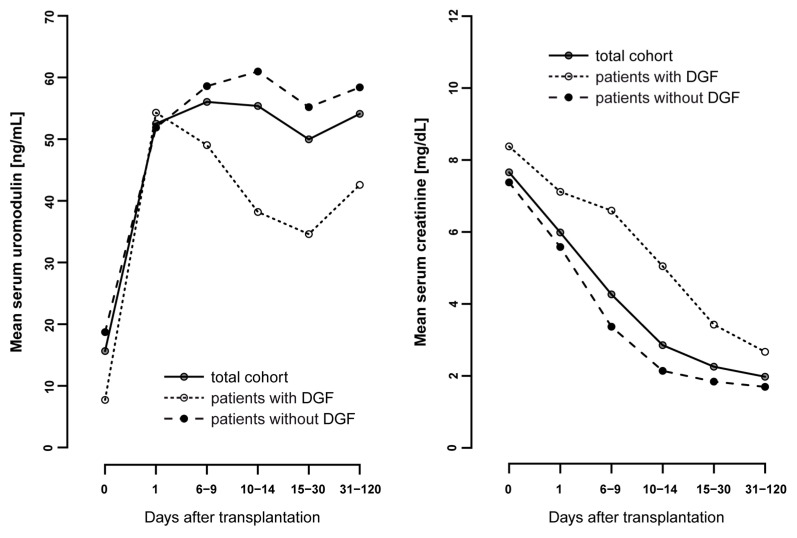
Mean serum uromodulin values [ng/mL] from pretransplant to follow-up (up to 120 days after transplantation) compared to the mean serum creatinine [mg/dL] in the total cohort and in patients with and without delayed graft function (DGF).

The mean sUMOD levels in the total cohort was 14.9 ± 23.8 ng/mL preoperatively, 52.3 ± 50.2 ng/mL on POD1 and remained stable after this up to 31–120 days after transplantation (Figure 1). Patients with DGF had significantly lower pretransplant sUMOD levels compared to patients without DGF (5.9 ± 6.4 ng/mL vs. 18.3 ± 26.8 ng/mL, *p* < 0.001). There was no significant difference in sUMOD levels on POD1 between patients with and without DGF (54.0 ± 50.4 ng/mL vs. 51.7 ± 50.3 ng/mL, *p* = 0.888; Table 1). However, while sUMOD levels decreased again in patients with DGF in the postoperative period, we did see a further increase in patients without DGF (Figure 1). In contrast, serum creatinine levels were higher in the DGF subgroup pretransplant and remained higher over the whole postoperative period compared to the non-DGF subgroup (Figure 1).

### 3.3. Pretransplant sUMOD and DGF

In univariable analysis, each 10 ng/mL higher preoperative sUMOD was associated with 49% lower odds for DGF (OR 0.51, 95%-CI 0.32–0.73, Table 3). This association remained statistically significant after multivariable adjustment (OR 0.53, 95%-CI 0.30–0.82). When categorized into quartiles, the quartile with the lowest preoperative sUMOD levels had 4.4-fold higher odds for DGF compared to the highest quartile in multivariable analysis (OR 4.41, 95%-CI 1.54–13.93, Table 3).

In order to rule out potential confounding through preemptive transplantation we performed a sensitivity analysis in which we adjusted our multivariable logistic regression Model 1 for preemptive transplantation (categorical variable yes vs. no) instead of dialysis vintage. We did not add preemptive transplantation as another covariable in order to avoid overfitting of the model. In this additional analysis with pretransplant sUMOD as a continuous variable, we identified a similar OR for the association of sUMOD with DGF (OR 0.50 (95%-CI 0.28–0.79) per 10 ng/mL higher sUMOD).

sUMOD on POD1 was not significantly associated with DGF, neither as a continuous nor a categorical variable (Table 3).

### 3.4. ROC-Analysis to Evaluate Preoperative sUMOD as a Predictor for DGF

**Figure 2:**
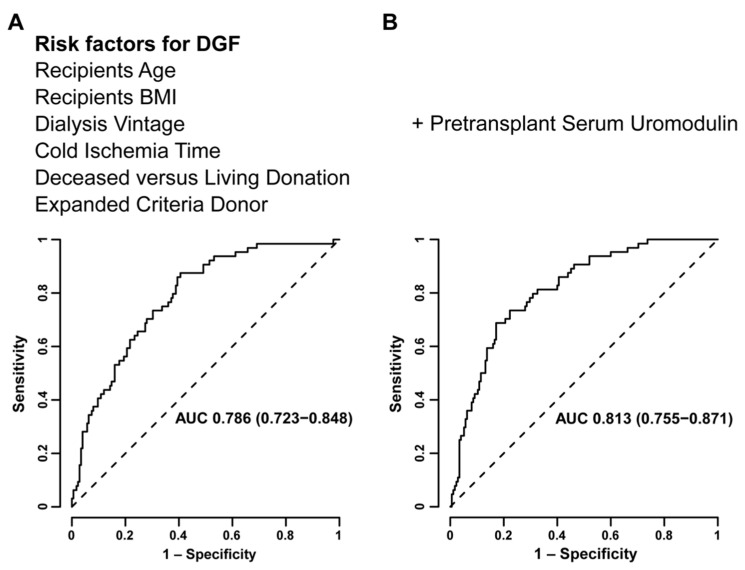
Multivariable receiver-operating-characteristic (ROC) curve analysis evaluating models including established risk factors (recipient age and body-mass-index (BMI), dialysis vintage, cold ischemia time, deceased vs. living donation, expanded criteria donors) for the prediction of delayed graft function (DGF) without (**A**) and with preoperative serum uromodulin (**B**).

In multivariable ROC curve analysis, Model A (including risk factors for DGF without preoperative sUMOD) worked moderately well to predict DGF (area under the curve (AUC) 0.786 [95%-CI 0.723–0.848], Figure 2). Model B (i.e., adding sUMOD to Model A) increased the predictive accuracy at borderline statistical significance (AUC 0.813 [95%-CI 0.755–0.871], *p* = 0.05) as presented in Figure 2.

## 4. Discussion

In the current study, we demonstrate that a higher pretransplant sUMOD in kidney transplant recipients is independently associated with a lower risk for DGF. Furthermore, preoperative sUMOD was of additional predictive value when added to a model of established risk factors for DGF. Surprisingly, we detected no association between sUMOD on POD1 and DGF.

We further mapped the course of sUMOD before, during and in the early phase after transplantation (up to 120 days following kidney transplantation) with and without the occurrence of DGF. We demonstrated that over the longer course after transplantation patients without DGF maintained higher sUMOD levels, while in patients with DGF we detected a subsequent decline in sUMOD in the postoperative period. The subsequent decrease in sUMOD is consistent with a recent study showing decrease in circulating uromodulin following AKI in a cohort of liver transplant patients undergoing surgery [26], reflecting tubular mass and function in the longer-term, non-acute setting.

sUMOD has been positively associated with reduced risk for kidney failure, cardiovascular events and mortality in geriatric and chronic kidney disease populations [27,28]. In kidney transplant setting, higher sUMOD in the first year after transplantation has also been associated with better long-term allograft survival in kidney transplant recipients [20,21]. Further, decreased concentrations of sUMOD can be observed in the early course of tubulointerstitial injury in the kidney transplant [29]. This is in line with our observations, that a higher pretransplant sUMOD is associated with lower risk for DGF due to IRI and subsequently higher sUMOD levels over the longer-term course following renal transplantation. None of the previous studies performed uromodulin measurements just before and after kidney transplantation.

Higher pretransplant sUMOD could represent the anti-inflammatory capacity of the recipient towards the following inflammation due to IRI. Interstitial or sUMOD has been shown to downregulate proinflammatory signaling in the kidney, reflecting its immunomodulatory and reno-protective capacity [30]. Recently, it was demonstrated that uromodulin inhibits the generation of reactive oxygen species both in the kidney and systemically [26]. In line with this, UMOD deficient mice experiencing IRI are at higher risk for acute kidney injury with higher interstitial inflammation and cell infiltration [17,19]. Furthermore, UMOD deficient mice showed delayed and incomplete recovery from acute kidney injury after IRI, which is explained by a lack of upregulation of uromodulin expression after IRI [14].

Although it is challenging to directly extrapolate results from murine models of IRI to human transplantation, results from these models support our observations, that a higher sUMOD in the recipient just before transplantation is associated with lower risk for DGF [31]. SUMOD is hypothesized to be a molecule with abilities in modulating inflammation against an evolving IRI, which in turn is thought to be one of the main mechanisms predisposing to DGF [32]. The findings that higher levels of preoperative sUMOD are associated with less risk of DGF leads to the hypothesis that there is a “high uromodulin” state before transplantation may be beneficial. However, given the observational nature of this data, we cannot conclude on whether sUMOD has a causal role to play in the development of DGF. Despite we detected significant differences in sUMOD levels between patients with and without DGF, absolute differences appear to be small compared to differences in sUMOD levels between healthy individuals and patients at different CKD stages [16]. Therefore, it remains to be validated that the differences we detected between DGF and non-DGF translate into physiologically relevant differences in uromodulin activity.

It is interesting that sUMOD increases initially in patients with or without DGF, which might reflect the release of “donor” sUMOD from the transplanted kidney. Patients with DGF have a subsequent profound and persistent decrease in sUMOD. The fact that we do not see an association between sUMOD on POD1 and delayed graft function could reflect the dynamic pathophysiological process occurring during this early time period in the transplanted kidney, which may be critical to the subsequent course of injury or recovery. The initial increase could represent general reactive reno-protection-intended induction of uromodulin production in the setting of renal IRI, which is related to its immune-modulatory capacities in the interstitium [14,17].

While sUMOD on POD1 might be influenced by acute inflammation and hypoxic stress, long-term sUMOD should reflect tubular function/mass [20,21]. However, as the primary aim of our study was to evaluate sUMOD as a predictive marker or a marker for early detection of DGF, sUMOD pre-transplant and on POD1 was the focus of our statistical analysis.

One strength of our study is the use of both pre- as well as the post-transplant period. While we did not directly adjust for residual kidney function in the multivariable approach, we propose that with adjusting for dialysis vintage and kidney transplantation after living donation we also captured residual kidney function to some extent, as it is well known that residual kidney function decreases along with the time spent of dialysis. In general, due to its molecular mass of 95 kDa sUMOD is highly unlikely to be removed by both hemo- and peritoneal dialysis.

A major limitation in the present study is that patients without DGF received kidneys from “healthier” donors with shorter ischemia time (see Table 2), that are less vulnerable to tubular injury. Although, we tried to account for this difference by adjusting for a number of covariables, which are supposed to be relevant risk factors for DGF (i.e., ECD, deceased vs. living donation, CIT) [2,5,25] there remains the potential residual confounding. Furthermore, DGF due to renal IRI is a common problem after deceased donation [2], but the proportion of patients after living donation in the present cohort is relatively high at almost 38%. We included transplant patients both after deceased as well as after living donation due to the otherwise small number of patients in a single center analysis. Further, we adjusted for living donation in statistical analysis as mentioned above. However, even after adjustment for deceased vs. living donation as well as dialysis vintage with expected shorter dialysis time before transplantation after living donation due to the large proportion of preemptive transplantations, recipients pretransplant sUMOD was independently associated with lower risk for DGF following transplantation. Finally, we lack data on single nucleotide polymorphisms (SNPs) that are known to influence uromodulin concentrations [33,34], and therefore, cannot comment on how these SNPs may affect our findings.

## 5. Conclusions

In conclusion, lower pretransplant sUMOD is independently associated with DGF after kidney transplantation and might therefore function as an early non-invasive marker to identify patients at increased risk for DGF following IRI and subsequent complicated course after kidney transplantation.

## Figures and Tables

**Table 1:** Overall baseline characteristics (n = 239) and baseline characteristics of participants stratified by quartiles distributed according to pretransplant serum uromodulin (sUMOD).

Characteristics	Total	Quartile 1 sUMOD: <2.59 ng/mL	Quartile 2 sUMOD: 2.59–7.04 ng/mL	Quartile 3 sUMOD: >7.04–14.66 ng/mL	Quartile 4 sUMOD: >14.66 ng/mL	*p*-Value
Number (no.) of patients	239	60	60	59	60	
Recipient demographics						
Age [years]	51 ± 14	50 ± 14	54 ± 13	52 ± 14	49 ±16	0.185
Female, no. (%)	75 (31.4)	17 (28.3)	20 (33.3)	15 (25.4)	23 (38.3)	0.443
Body-mass index [kg/m^2^]	25.3 ± 4.8	25.0 ± 5.1	25.6 ± 5.1	26.0 ± 4.8	24.8 ± 4.3	0.525
Diabetes, no. (%)	48 (20.1)	4 (6.7)	18 (30.0)	15 (25.4)	11 (18.3)	0.009
Hypertension, no. (%)	194 (81.2)	46 (76.7)	51 (85.0)	50 (84.7)	47 (78.3)	0.536
Cardiovascular disease, no. (%)	79 (33.1)	18 (30.0)	25 (41.7)	22 (37.3)	14 (23.3)	0.151
Dialysis vintage [days]	1559 ± 1418	2137 (1469)	1921 ± 1392	1220 ± 1267	953 ± 1215	<0.001
Preemptive transplant, no. (%)	29 (12.1)	3 (5.0)	1 (1.7)	7 (11.9)	18 (30.0)	<0.001
Pretransplant sUMOD [ng/mL]	14.9 ± 23.8	0.9 ± 0.8	4.5 ± 1.3	10.1 ± 2.0	44.2 ± 32.8	<0.001
Recipient laboratory measures on postoperative Day 1 (POD1)						
sUMOD [ng/mL]	52.3 ± 50.2	56.0 ± 65.1	50.6 ± 51.1	36.4 ± 24.5	65.3 ± 48.7	0.014
Serum creatinine [mg/dL]	6.0 ± 2.3	6.0 ± 2.1	6.7 ± 2.4	6.1 ± 2.4	5.2 ± 2.2	0.004
Hemoglobin [g/dL]	10.3 ± 1.6	10.3 ± 1.8	10.6 ± 1.6)	10.5 ± 1.4	10.0 ± 1.6	0.223
Leucocyte count [G/L]	11.6 ± 4.2	10.6 ± 3.8	11.7 ± 3.8	11.6 ± 4.5	12.6 ± 4.5	0.095
C-reactive protein [mg/dL]	3.4 ± 2.3	2.6 ± 1.4	6.0 ± 3.6	3.2 ± 1.7	2.8 ± 1.6	<0.001
Sodium [mmol/L]	141 ± 4	140 ± 4	141 ± 4	141± 5	141 ± 4	0.093
Potassium [mmol/L]	4.9 ± 0.8	5.1 ± 0.7	5.0 ± 0.8	4.7 ± 0.7	4.6 ± 0.8	<0.001
Donor characteristics						
Age [years]	54.4 ±15.5	51 ± 16	55 ±16)	55 ± 15)	52 ± 15	0.256
Female, no. (%)	118 (49.4)	25 (41.7)	32 (53.3)	29 (49.2)	32 (53.3)	0.536
Body-mass index [kg/m^2^]	26.4 ± 4.4	27.0 ± 5.2	26.0 ± 3.7	26.5 ± 4.5	26.1 ± 3.8	0.574
Diabetes, no. (%)						0.131
No	166 (69.5)	37 (61.7)	46 (76.7)	35 (59.3)	48 (80.0)	
Yes	15 (6.3)	4 (6.7)	4 (6.7)	5 (8.5)	2 (3.3)	
Unknown	58 (24.3)	19 (31.7)	10 (16.7)	19 (32.2)	10 (16.7)	
Hypertension, no. (%)						0.071
No	119 (49.8)	29 (48.3)	30 (50.0)	23 (39.0)	37 (61.7)	
Yes	79 (33.1)	16 (26.7)	22 (36.7)	27 (45.8)	14 (23.3)	
Unknown	41 (17.2)	15 (25.0)	8 (13.3)	9 (15.3)	9 (15.0)	
Serum creatinine [mg/dL]	1.0 ± 0.7	1.0 ± 0.8	0.9 ± 0.5)	1.1 ± 0.8	1.0 ± 0.7	0.740
Expanded criteria donor, no (%)	97 (40.6)	20 (33.3)	28 (46.7)	28 (47.5)	21 (35.0)	0.245
Transplant related variables						
Living donation, no. (%)	90 (37.7)	14 (23.3)	14 (23.3)	27 (45.8)	35 (58.3)	<0.001
Cold ischemic time [hours]	8 ± 6	10 ± 6	10 ± 6	7 ± 5	6 ± 6	<0.001
Warm ischemic time [minutes]	25 ± 13	26 ± 12	27 ± 13	27 ± 16	23 ± 7	0.313
Primary non-function, no. (%)	8 (3.3)	1 (1.7)	2 (3.3)	3 (5.1)	2 (3.3)	0.783
No. of HLA-mismatches	4 ± 2	4 ± 2	4 ± 2	3 ± 2	3 ± 2	0.410

Continuous variables presented as mean ± standard deviation, categorical variables presented in percentage of referring population. The *p*-value will compare variables between quartiles calculated by parametric testing. sUMOD, serum uromodulin.

**Table 2:** Baseline characteristics of participants stratified by status regarding delayed graft function (DGF).

Characteristics	Without DGF	With DGF	*p*-Value
Number (no.) of patients	175	64	
Recipient demographics			
Age [years]	50 ± 14	56 ± 13	0.003
Female, no. (%)	61 (34.9)	14 (21.9)	0.079
Body-mass index [kg/m^2^]	24.5 ± 4.4	27.8 ± 5.2	<0.001
Diabetes, no. (%)	30 (17.1)	18 (28.1)	0.090
Hypertension, no. (%)	141 (80.6)	53 (82.8)	0.837
Cardiovascular disease, no. (%)	45 (25.7)	34 (53.1)	<0.001
Dialysis vintage [days]	1321 ± 1331	2208 ± 1456	<0.001
Preemptive transplant, no. (%)	28 (16.0)	1 (1.6)	<0.001
Pretransplant sUMOD [ng/mL]	18.3 ± 26.8	5.9 ± 6.4	<0.001
Recipient laboratory measures on postoperative Day 1 (POD1)			
sUMOD [ng/mL]	51.7 ± 50.3	54.0 ± 50.4	0.747
Serum creatinine [mg/dL]	5.6 ± 2.2	7.1 ± 2.4	<0.001
Hemoglobin [g/dL]	10.3 ± 1.6	10.4 ± 1.8	0.687
Leucocyte count [G/L]	11.5 ± 4.2	12.0 ± 4.2	0.479
C-reactive protein [mg/dL]	3.5 ± 2.6	3.2 ± 1.3	0.585
Sodium [mmol/L]	141 ± 4	139 ± 5	0.005
Potassium [mmol/L]	4.7 ± 0.7	5.4 ± 0.6	<0.001
Donor characteristics			
Age [years]	52 ± 15	57 ± 15	0.021
Female, no. (%)	96 (54.9)	22 (34.4)	0.008
Body-mass index [kg/m^2^]	25.8 ± 3.7	28.1 ± 5.5	<0.001
Diabetes, no. (%)			<0.001
No	131 (74.9)	35 (54.7)	
Yes	5 (2.9)	10 (15.6)	
Unknown	39 (22.3)	19 (29.7)	
Hypertension, no. (%)			0.209
No	93 (53.1)	26 (40.6)	
Yes	55 (31.4)	24 (37.5)	
Unknown	27 (15.4)	14 (21.9)	
Serum creatinine [mg/dL]	1.0 ± 0.6	1.1 ± 0.9	0.057
Expanded criteria donor, no (%)	64 (36.6)	33 (51.6)	0.052
Transplant related variables			
Living donation, no. (%)	79 (45.1)	11 (17.2)	<0.001
Cold ischemic time [hours]	7.2 ± 6.0	9.9 ± 5.5	0.002
Warm ischemic time [minutes]	24 ± 12	29 ± 14	0.006
Primary non-function, no. (%)	0 (0)	8 (12.5)	<0.001
No. of HLA-mismatches	3 ± 2	3 ± 2	0.650

Continuous variables presented as means ± standard deviation, categorical variables presented in percentage of referring population. The *p*-value will compare recipients with DGF and without DGF calculated by parametric testing. sUMOD, serum uromodulin.

**Table 3:** Associations of serum uromodulin (sUMOD) pretransplant and on postoperative Day 1 with delayed graft function (DGF) in the kidney transplant.

	Events	Unadjusted	Model 1 ^a^	Model 2 ^b^	Model 3 ^c^
**Pretransplant sUMOD**					
Per 10 ng/mL higher sUMOD	64/239	0.51 (0.32–0.73)	0.54 (0.31–0.81)	0.55 (0.31–0.83)	0.53 (0.30–0.82)
Q1	25/60	5.41 (2.21–14.80)	4.47 (1.62–13.61)	4.30 (1.53–13.31)	4.41 (1.54–13.93)
Q2	20/60	3.79 (1.52–10.46)	2.55 (0.93–7.61)	1.94 (0.68–5.93)	1.95 (0.67–6.08)
Q3	12/59	1.93 (0.72–5.58)	1.52 (0.52–4.70)	1.28 (0.42–4.06)	1.29 (0.42–4.14)
Q4	7/60	1 (ref.)	1 (ref.)	1 (ref.)	1 (ref.)
**sUMOD on postoperative Day 1**					
Per 10 ng/mL higher sUMOD	63/237 *	1.01 (0.95–1.07)	1.01 (0.95–1.07)	1.03 (0.96–1.09)	1.03 (0.96–1.09)
Q1	16/60	0.90 (0.40–2.01)	0.71 (0.28–1.75)	0.70 (0.27–1.78)	0.71 (0.27–1.86)
Q2	14/59	0.77 (0.33–1.74)	0.72 (0.27–1.85)	0.77 (0.29–2.03)	0.79 (0.29–2.14)
Q3	16/59	0.92 (0.41–2.06)	0.84 (0.34–2.07)	0.88 (0.35–2.22)	0.86 (0.34–2.17)
Q4	17/59	1 (ref.)	1 (ref.)	1 (ref.)	1 (ref.)

Results are presented as odds ratios with 95%-confidence intervals given in parentheses. Delayed graft function is defined as the requirement of >1 dialysis treatment within the first week after transplantation. Quartile distribution according to preoperative serum uromodulin (sUMOD): Quartile 1 (Q1) ≤ 2.59 ng/mL, Quartile 2 (Q2) > 2.59–7.04 ng/mL, Quartile 3 (Q3) > 7.04–14.66 ng/mL, Quartile 4 (Q4) > 14.66 ng/mL. Quartile distribution according to postoperative Day 1 serum uromodulin (sUMOD): Quartile 1 (Q1) ≤ 22.00 ng/mL, Quartile 2 (Q2) > 22.00–36.97 ng/mL, Quartile 3 (Q3) > 36.97–68.44 ng/mL, Quartile 4 (Q4) > 68.44 ng/mL. * Two patients missing due to missing sUMOD values on postoperative Day 1. ^a^ Adjusted for recipients age, recipients body-mass-index and dialysis vintage. ^b^ Model 1 + serum creatinine on postoperative Day 1. ^c^ Model 2 + cold-ischemia time, living vs. deceased donor transplantation, expanded criteria donors (ECD).

## Data Availability

The dataset generated during the current study is available from corresponding author upon reasonable request.

## List of Abbreviations

AUC	Area under the curve
BMI	Body-mass index
CIT	Cold ischemia time
ECD	Expanded criteria donor
eGFR	Estimated glomerular filtration rate
ELISA	Enzyme-linked immunosorbent assay
DGF	Delayed graft function
IRI	Ischemia-reperfusion injury
95%-CI	95% confidence interval
OR	Odds ratio
POD1	Postoperative Day 1
ROC	Receiver-operating-characteristics
sUMOD	Serum uromodulin.
